# Hearing loss and its association with all-cause and cause-specific mortality: A meta-analysis of cohort studies

**DOI:** 10.1371/journal.pone.0333125

**Published:** 2025-10-09

**Authors:** Jinli Jia, Hongyan Li, Chunlan Xu

**Affiliations:** 1 Department of Nursing, Beijing Tiantan Hospital, Capital Medical University, Beijing, China; 2 Department of Otorhinolaryngology Head and Neck Surgery, Beijing Tiantan Hospital, Capital Medical University, Beijing, China; Gaziantep Islam Science and Technology University, Medical Faculty, Division of Pediatric Gastroenterology, TÜRKIYE

## Abstract

**Background:**

This study aimed to examine the epidemiological associations between hearing loss and the risk of all-cause mortality, cardiovascular mortality, and cancer mortality.

**Methods:**

A comprehensive search was performed in PubMed, Cochrane Library, and Embase for cohort studies published from database inception to January 12, 2025, using relevant MeSH terms and keywords. Study quality was assessed using the Newcastle-Ottawa Scale (NOS). A random-effects model was applied for the meta-analysis. Sensitivity analysis was conducted by sequentially excluding individual studies to assess the robustness of the results. Subgroup analyses were performed based on hearing function measurement methods, study design, and continent. Publication bias was evaluated using funnel plots and Egger’s test. Statistical analyses were conducted using Stata 14.0.

**Results:**

This meta-analysis included 36 cohort studies with a total of 6,364,914 participants, published between 1992 and 2024. Hearing loss was significantly associated with all-cause mortality (HR: 1.21; 95% CI: 1.13–1.31; *I²* = 95.7%, **P* *< 0.001), cardiovascular mortality (HR: 1.22; 95% CI: 1.12–1.33; *I²* = 52.4%, *P* < 0.001), and cancer mortality (HR: 1.11; 95% CI: 1.02–1.22; *I²* = 51.2%, **P* *= 0.016), after adjusting for demographics and comorbidities. Subgroup analysis showed that audiometrically measured hearing loss had a stronger effect than self-reported hearing loss (HR: 1.28; 95% CI: 1.10–1.49; *I²* = 97.7%, *P* = 0.002). The risk of all-cause mortality was higher in prospective cohorts compared to retrospective cohorts (HR: 1.24; 95% CI: 1.05–1.46; *I²* = 97.2%, *P* = 0.012). Additionally, the risk of all-cause mortality was slightly higher in studies from Asia compared to other continents (HR: 1.33; 95% CI: 1.09–1.62; *I²* = 98.3%, **P* *= 0.005).

**Conclusions:**

Our meta-analysis indicates that hearing loss is associated with an increased risk of all-cause, cardiovascular, and cancer mortality. Healthcare providers managing patients with hearing loss should consider its potential impact on overall health and longevity.

**PROSPERO registration number:**

CRD 42025637635

## Introduction

Hearing loss (HL) represents a major global health issue, affecting more than 1.55 billion people worldwide in 2021 [1], with projections indicating that this number will rise to 2.5 billion by 2050 [2]. Unaddressed hearing loss costs the global economy approximately $980 billion annually, including healthcare, education, productivity losses, and societal costs [3]. Despite its widespread prevalence, HL has often been overlooked in public health priorities, partly due to its gradual onset and the subtle progression of auditory function [4]. Recent evidence, however, suggests a significant association between HL and an increased risk of mortality in adults [5,6]. This association may be mediated through various factors, including cognitive decline [7], walking disability, fall-related injuries [8], cardiovascular diseases [9], frailty [10] and social isolation [11]. However, some studies have reported no such association [12]. While previous meta-analyses [13] have explored this issue, most earlier studies primarily focused on all-cause mortality, with limited examination of cause-specific risks, such as cardiovascular or cancer-related mortality. Additionally, few reviews have addressed potential geographic or study design differences, which may impact the generalizability of their findings.

To fill these gaps, we present an updated meta-analysis that includes 36 cohort studies encompassing over 6.4 million participants. By synthesizing global evidence and conducting subgroup analyses based on measurement methods, study types, and geographic regions, this study aims to clarify the strength and consistency of the relationship between HL and mortality, offering valuable insights for clinical and public health interventions.

## Methods

This meta-analysis followed the Preferred Reporting Items for Systematic Reviews and Meta-Analyses (PRISMA) guidelines [14]. The protocol was pre-registered on the PROSPERO platform (registration number: CRD 42025637635) on January 13,2025.

### Data sources and searches

We conducted a comprehensive search of PubMed, Embase, and Cochrane Library from inception to January 12, 2025, with no language restrictions. The search strategy combined MeSH terms and keywords related to HL and mortality. The full PubMed search strategy is detailed in S1 Table. We also reviewed reference lists of included studies and relevant meta-analyses to identify additional studies.

### Eligibility criteria

We included cohort studies that examined the association between HL and all-cause or cause-specific mortality risk among adults aged 18 years and older. Studies were excluded if they met any of the following criteria: ①Did not report hazard ratios (HRs) with 95% confidence intervals (CIs) for mortality outcomes.②They were conference abstracts, study protocols, duplicate publications, or studies without relevant outcomes. ③Duplicate publications or sub-studies of the same cohort (only the most comprehensive study was retained). ④ Focused on specific subpopulations (e.g., cancer patients, post-stroke survivors) where HL was not the primary exposure.

### Study selection

Two authors independently selected eligible studies (based on title and abstract, followed by full-text articles), extracted relevant data, and evaluated the risk of bias in a blinded manner, with conflicts resolved by a third author.

Study selection was performed by two reviewers (JLJ and CLX) who independently screened the literature based on the eligibility and exclusion criteria. Duplicate and irrelevant articles were first excluded according to their titles and abstracts. Thereafter, the full texts of the potentially eligible articles were downloaded and read to identify all eligible studies. Any disagreements were resolved by the third reviewer (HYL), who acted as an arbiter.The main reasons for excluding studies is displayed in S2 Table.

### Data extraction

Data were independently extracted by two reviewers using pre-designed forms. Extracted data included study details (author, year, country, sample size, follow-up duration), HL definition, outcomes, and adjusted confounders. Disagreements were resolved by consensus with the third reviewer.

### Risk of bias

The Newcastle-Ottawa Scale (NOS) [15] was used to assess study quality. Studies were rated on a scale of 0–9 stars, with higher scores indicating better quality. Scores of 0–3, 4–6, and 7–9 were considered low, moderate, and high quality, respectively.

### Statistical analysis

We used adjusted HRs and 95% CIs to assess the association between HL and mortality risk. Heterogeneity was evaluated using the χ² test and I² values. Given the potential for both statistical and clinical heterogeneity, a random-effects model was applied [16,17]. Sensitivity analysis was conducted by excluding one study at a time to test the robustness of the findings. Statistical significance was defined as a two-sided P-value < 0.05 for all analyses. Funnel plots were visually inspected for publication bias, and Egger’s test was used for statistical assessment. Subgroup analyses were performed based on hearing assessment methods, study type, and continent. All statistical analyses were conducted using Stata 14.0.

## Results

### Literature search

**Fig 1:**
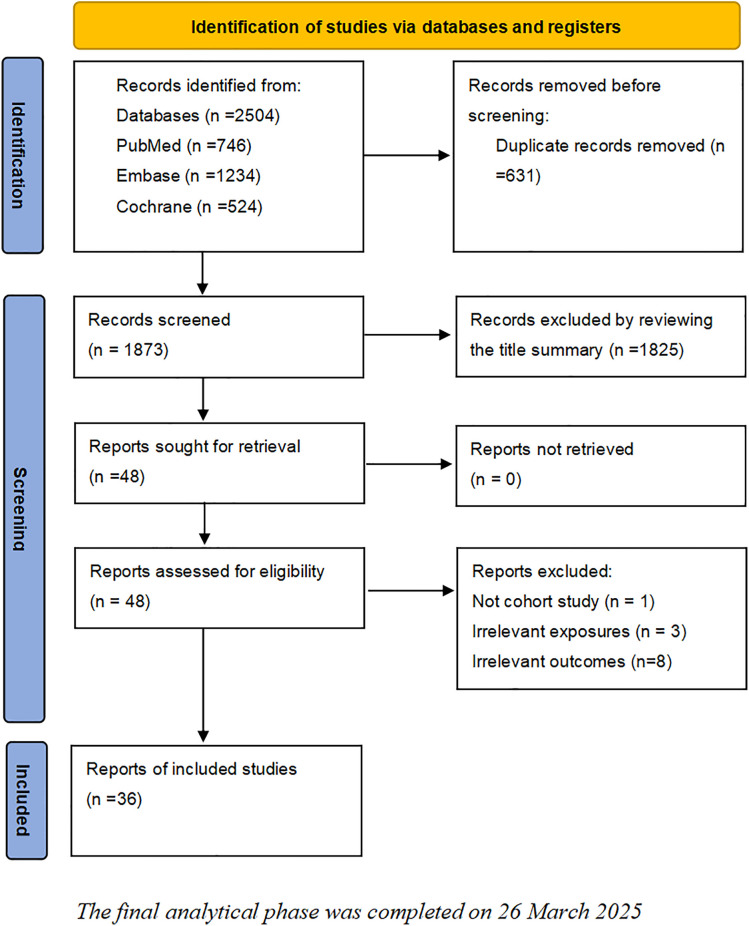
Flow chart of literature screening.

The systematic search of cohort studies published before January 12, 2025, identified 2504 results. After title and abstract screening, 48 articles were considered potentially relevant. Thirty-six studies were included after full text review, of which 35 studies reported association between HL and all-cause mortality, 10 studies reported association between HL and cardiovascular mortality, and 6 studies reported association between HL and cancer mortality, The selection process is presented in Fig 1.

### Study characteristics

Of the 36 included studies [5–7,9,12,18–48] all were observational, with 13 retrospective cohorts [5,7,18,21,26,29,36,37,43,45–48], 23 prospective cohorts [6,9,12,19,20,22–25,27,28,30–35,38–42,44]. When assessed using the NOS, 26 studies [5–7,9,12,18–22,24,26–32,35–37,39,41–44] and 10 studies [23,25,33,34,37,38,40,45–48] had a high and moderate risk of bias, respectively. The scores of the included studies are shown in S3 Table. Sensitivity analyses excluding the studies with a high risk of bias did not substantially alter our results. A total of 14 [5,6,9,18,29,32,33,35,37,41,43–46], 14 [12,19–21,23–28,38,39,42,48], 5 [22,30,31,34,47], and 3 studies [7,36,40] were conducted in North America, Asia, Europe, and Oceania, respectively. We included 36 studies in our meta-analysis. The main characteristics of the included trials are shown in Table 1.

**Table 1:** Basic characteristics of the included studies.

Author	Year	Country	Study type	Sample size	Follow-up years	Definition of HL	Outcomes	Confounders adjusted
Dement J[5]	2024	USA	Retrospective cohort study	19379	11.1 medians	Audiometry: HL was defined as a pure‐tone average HL > 25dB in the better hearing ear	All-cause mortality	Age, Sex, gender, race/ethnicity, smoking, medical histories of hypertension, diabetes, cardiovascular disease, personal cancer history, dementia, and heavy alcohol consumption, COPD by spirometry, chest X-ray parenchymal profusion≥1/0, and a covariate for work in a construction trade
Choi JS [6]	2024	USA	Prospective cohort study	9885	10.4 medians	Audiometry: HL was defined as speech- frequency pure-tone average at 25 dB HL (hearing level) or greater in the better hearing ear	All-cause mortality	Age, sex, race/ethnicity, education, poverty-to-income ratio, marital status, health insurance, smoking, BMI, diabetes, hypertension, cardiovascular disease, and stroke
Zhang H[12]	2023	China	Prospective cohort study	18625	5.5 averages	Audiometry: Pure-tone air- conduction audiometry was performed to measure the hearing thresholds at five frequencies (0.5, 1, 2, 4, and 8 kHz) over an intensity range of−10–120 dB in a sound- treated booth using the Micro-DS PZD-21 audiometer	All-cause mortality Cardiovascular mortality	Age, gender, BMI, marital status, education levels, physical activity, smoking status, drinking status, T2DM, hypertension, hyperlipidemia, family history of CVD and occupational noise exposure and occupational noise exposure
Tonelli M[18]	2023	Canada	Retrospective cohort study	4724646	14.4 medians	Audiometry: created an algorithm to identify participants with HL using provider claims, at least two claims 30 days apart. a second algorithm for HL using one hospitalization or two provider claims within 2 years.	All-cause mortality	Rural residence status, material deprivation quintiles, obesity, hypertension, chronic pain, depression, chronic pulmonary disease, diabetes mellitus, hypothyroidism, osteoporosis, gout, stroke or TIA, fragility fractures, heart failure, cancer, asthma, alcohol misuse, coronary artery disease, atrial fibrillation, irritable bowel syndrome, rheumatic diseases, epilepsy, dementia, schizophrenia, inflammatory bowel disease, multiple sclerosis, severe constipation, peripheral artery disease, Parkinson’s disease, psoriasis, severe chronic kidney disease, pepticulcer disease, and chronic liver disease
Xu S[19]	2023	China	Prospective cohort study	120818	9 medians	Audiometry: determined (i.e., speech-in-noise test), Self-reported hearing problems	Cardiovascular mortality Cancer mortality	Ethnicity, annual household income, smoking and alcohol intake, exposure to working noise, BMI
Feng X[20]	2022	China	Prospective cohort study	11732	12.15 medians	Audiometry measurement	All-cause mortality Cardiovascular mortality Cancer mortality	Age, gender, race, educational levels, BMI, family income-poverty ratio level, married status, smoking status, drinking status, physical activity, general health condition, healthy eating index, occupational noise exposure, history of diabetes, cardiovascular disease, hypertension, cancer
Cui Y[21]	2022	China	Retrospective cohort study	455988	Not stated	Self-report: HL status was ascertained at the initial interview via two binaries coded	All-cause mortality Cardiovascular mortality Cancer mortality	Education, physical activity, smokers, Alcohol intakes, BMI, hypertension, diabetes
Langballe EM [22]	2022	Norway	Prospective cohort study	30080	35	Self-reported and based on the initial yes/no question	All-cause mortality	Age, sex, education, work and living situation, smoking status at baseline, BMI, SBP
Wang J[23]	2022	China	Prospective cohort study	10744	8	Self-report: using a dichotomized metric of self-reported hearing status	All-cause mortality	Gender, age, education attainment, residence, marital status, smoking status, drinking status, regular leisure activities, ADL, hypertension, diabetes, cognitive function
Kim SY [24]	2020	Korea	Prospective cohort study	28065	6.9 averages	Audiometry: be tested three times using the PTA and one time with the auditory brainstem response threshold test	All-cause mortality	Age, sex, income, region of residence, hypertension, diabetes, dyslipidemia, ischemic heart disease, stroke histories
Sun J[25]	2020	China	Prospective cohort study	37076	Not stated	Self-report	All-cause mortality	Age, sex, enrollment year, province, residence, ethic, marriage status, occupation, access to medical service, smoking status, drinking status, exercise status, ADL score, physical performance score, MMSE score, food diversity score, social activity score, and chronic disease score
Han HM [26]	2020	Korea	Retrospective cohort study	160205	Not stated	Audiometry: under go at least three tests of pure tone audiometry (PTA) exams within a 2–7-day interval. The PTA results must then be confirmed using an auditory brainstem response (ABR) or auditory steady-state response (ASSR).	All-cause mortality	Age, sex, smoking, alcohol consumption, regular exercise, hypertension, dyslipidemia, and diabetes mellitus
Zhang Y[27]	2020	China	Prospective cohort study	8788	2.5 medians	Self-report:the interviewers if participants could hear clearly what the interviewers said during the questionnaire survey	All-cause mortality	Age, sex, education years, ethnicity, marital status, co-residence, residence, economic independence, smoking status, drinking status, regular exercise, ADL disability, cognitive impairment, BMI and number of diseases
Miyawaki A[28]	2020	Japan	Prospective cohort study	9522	9 averages	Self-report	All-cause mortality Cardiovascular mortality Cancer mortality	Age, sex, education years, the living area, income level, marital status, primary occupation, self-rated health, self-reported histories of cancer, stroke, heart disease, diabetes, dyslipidemia, hypertension, body mass index, smoking status, exercise habits, alcohol consumption, dietary patterns, walking ability, depression, social participation
Lin HW [29]	2019	USA	Retrospective cohort study	123899	5	Self-report	All-cause mortality	Age, sex, ethnicity, race, comorbidity count (diabetes, cancer history, heart disease, stroke)
Engdahl B[30]	2019	Norway	Prospective cohort study	50462	17.6 averages	Pure-tone audiometry	All-cause mortality Cardiovascular mortality Cancer mortality	Smoking, alcohol use, physical activity, diabetes, resting heart rate, waist circumference, myocardial infarction, angina pectoris, stroke/brain haemorrhage, cancer, income, education, marriage, cohabiting, single and children status
Amieva H[31]	2018	France	Prospective cohort study	3777	25	Self-report	All-cause mortality	Age, sex, education, number of comorbidities (hypertension, myocardial infarction, angoranimi, diabetes, dyspnea, history of stroke, and smoking)
Schubert CR [32]	2017	USA	Prospective cohort study	2418	12.8 averages	Audiometry: pure-tone air and bone conduction audiometry	All-cause mortality	Age, sex, education, hypertension, diabetes, cardiovascular disease, cancer, cognitive impairment, frailty, smoking, exercise, BMI, alcoho, intima media thickness, C-reactive protein, and interleukin-6
Loprinzi PD [33]	2016	USA	Prospective cohort study	1658	7.67 medians	Self-report	All-cause mortality	Age, sex, race and ethnicity, HbA1c level, waist circumference, medications used to treat dizziness/ balance problems
Liljas AE [34]	2016	UK	Prospective cohort study	3981	10	Self-report	All-cause mortality	Age, social class, BMI, smoking, physical activity, cardiovascular diseases, hypertension, diabetes
Genther DJ [9]	2015	USA	Prospective cohort study	1958	8	Audiometry: defined as hearing level > 25dB for the PTA of 4 frequencies, in the better ear, according to WHO criteria	All-cause mortality	Age, race, sex, education, site, smoking, hypertension, diabetes, stroke, hearing aid use, pure-tone average mean, modified mini-mental state exam, gait speed, Center for Epidemiologic Studies Depression Scale score
Fisher D[35]	2014	USA	Prospective cohort study	4926	5	Audiometry: defined as hearing level ≥ 35dB for the PTA of 4 frequencies, in the better ear	All-cause mortality Cardiovascular mortality	Age, smoking status, BMI, hypertension, diabetes, systolic blood pressure, self-reported health status, cognitive status, self-reported history of falls, history of angina, record of cardiovascular event, hearing aid use
Gopinath B[36]	2013	Australia	Retrospective cohort study	2812	10	Audiometry: defined as hearing level > 25dB for the PTA of 4 frequencies, in the better ear, according to WHO criteria	All-cause mortality Cardiovascular mortality Cancer mortality	Age, sex, BMI, systolic blood pressure, current smoking status, poor self-rated health, walking disability, presence of hypertension and/or diabetes, history of cancer, angina, stroke and/or acute myocardial infarction, cognitive impairment
Feeny D[37]	2012	Canada	Retrospective cohort study	12375	12	Self-report	All-cause mortality	Sociodemographic factors (age, sex, marital status, household income, education), chronic conditions associated with high risk of mortality (high blood pressure, chronic bronchitis or emphysema, diabetes, heart disease, cancer, stroke), chronic conditions possibly associated with mortality (asthma, Alzheimer disease, other dementia), conditions not associated with high risk of mortality (food allergies, other allergies, arthritis or rheumatism, back problems excluding arthritis, migraine headaches, sinusitis, epilepsy, stomach or intestinal ulcers, urinary incontinence, cataracts, glaucoma, and other long-term conditions), BMI, health behaviors (smoking, physical activity, alcohol use), psychological health and resources (psychological distress, sense of coherence, chronic stress), perceived social support
Yamada M[38]	2011	Japan	Prospective cohort study	1250	3	Self-report	All-cause mortality	Age, sex, marital status, level of education, existence of support network, vision ability, depressed feeling, self-reported well-being, stroke, myocardial infarction, COPD, diabetes, rheumatoid arthritis; additional adjustment for hearing aids did not substantially change the association (HR NS)
Agrawal N[39]	2011	India	Prospective cohort study	1422	1.42medians	Audiometry: the Whisper test first and he Rinne’s test and Weber’s test, using a 512 Hz tuning fork by the investigator.	All-cause mortality	Age, sex, literacy, Hypertension, Diabetes mellitus, Coronary Artery Disease, Stroke, Orthopedic impairment, Dressing, Feeding, Self-Rated Health
Lopez D[40]	2011	Australia	Prospective cohort study	5354	6.36 averages	Self-report	All-cause mortality	NA
Karpa MJ [7]	2010	Australia	Retrospective cohort study	2956	5	Audiometry: defined as hearing level > 25dB for the PTA of 4 frequencies, in the better ear, according to WHO criteria	All-cause mortality Cardiovascular mortality	Age, sex, smoking, alcohol, hypertension, home ownership, low BMI, angina, acute myocardial infarction, walking disability, cognitive impairment, self-reported health, high serum urate
Denney JT [41]	2021	USA	Prospective cohort study	198902	11	Self-report	All-cause mortality	Age, sex, race, education level, household poverty, employment status, self-rated health, marital status, household composition
Lee W[42]	2020	Korea	Prospective cohort study	580798	8.4 medians	Audiometry: defined as hearing level > 25dB for the PTA of 4 frequencies, in the better ear, according to WHO criteria	All-cause mortality Cardiovascular mortality	Age, sex, study center, year of exam, smoking status, alcohol consumption, regular exercise, BMI, education level, exposure to occupational noise, diabetes, hypertension, cancer, cardiovascular disease, medication for dyslipidemia
Liu PL [43]	2016	USA	Retrospective cohort study	3871	6	Self-report	All-cause mortality	Age, sex, race, education, marital status, body mass index, current or past history of smoking, depression, and a weighted health index score of 5 chronic diseases (cancer excluding skin cancer, stroke, diabetes, hypertension, myocardial infarction)
Reuben DB [44]	1999	USA	Prospective cohort study	5444	10.1 averages	Audiometry: defined as> 40dB loss at either 1 or 2kHz or >40-dB loss at 1 and 2 kHz in both ears	All-cause mortality	Age, sex, race, education, myocardial infarction, diabetes, hypertension, heart failure, follow-up
Laforge RG [45]	1992	USA	Retrospective cohort study	1408	1	Self-report	All-cause mortality	Age, sex, cognitive dysfunction
Lam BL [46]	2006	USA	Retrospective cohort study	116796	7 averages	Self-report	All-cause mortality	Age, marital status, educational level, self-rated health, and number of nonocular and nonauditory conditions
Appollonio I[47]	1995	Italy	Retrospective cohort study	1140	6	Audiometry: whisper test	All-cause mortality	NA
Mitoku K[48]	2016	Japan	Retrospective cohort study	1754	4.7 averages	Self-report	All-cause mortality	Age, sex, level of dependency, diabetes, neurological disease, hypertension, heart disease, cerebrovascular disease, respiratory disease, musculoskeletal and connective tissue disease, eye and ear disease

### Association of HL and mortality

#### Meta-analysis for all-cause mortality.

**Fig 2:**
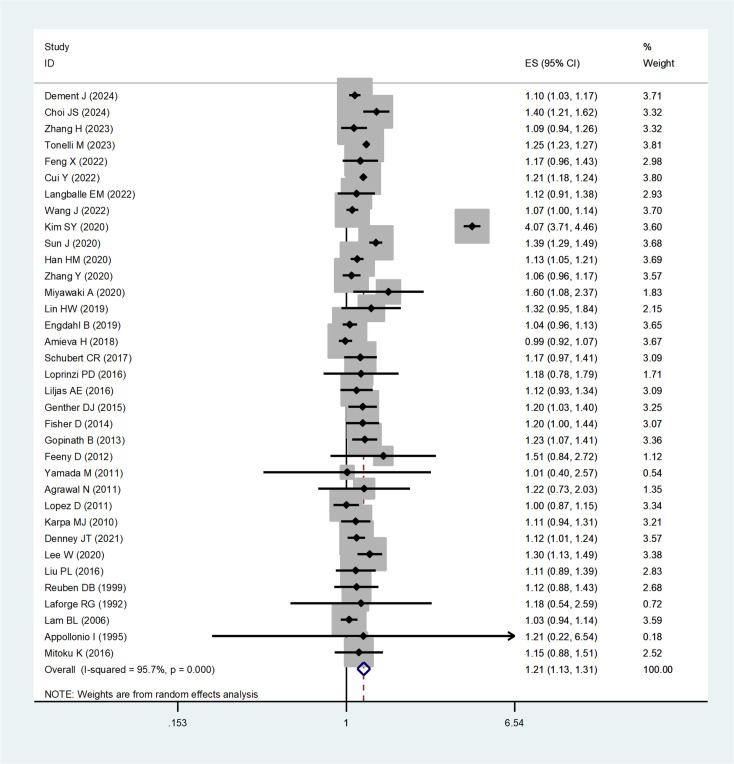
Forest plot for the the risk of all-cause mortality caused by HL.

Thirty-five studies were included in the analysis of all-cause mortality. Participants with HL had a significantly higher pooled hazard ratio (HR) for all-cause mortality compared to those with normal hearing (HR, 1.21; 95% CI, 1.13–1.31; I² = 95.7%, P = 0.000, Fig 2). Sensitivity analysis confirmed the robustness of the results (S1 Fig).

Subgroup analyses revealed slightly higher HRs for audiometric assessments (HR, 1.28; 95% CI, 1.10–1.49; I² = 97.7%, P = 0.002) and for prospective cohorts (HR, 1.24; 95% CI, 1.05–1.46; I² = 97.2%, P = 0.012). The risk of all-cause mortality was higher in studies conducted in Asia compared to other continents (Table 2).

**Table 2:** Subgroup analysis for the risk of all-cause mortality with HL.

Subgroups	Included studies	HR (95% CI)	Heterogeneity	
			*I*^*2*^ (%)	*P*-values
Assessment method				
Audiometry	17	1.28 (1.10-1.49)	97.7	0.000
Self-report	18	1.13 (1.06-1.20)	77.4	0.000
Study type				
Retrospective cohort	13	1.17 (1.12-1.22)	68.9	0.000
Prospective cohort	22	1.24 (1.05-1.46)	97.2	0.000
Area				
North America	14	1.17 (1.11-1.24)	65.7	0.000
Asia	13	1.33 (1.09-1.62)	98.3	0.000
Europe	5	1.03 (0.98-1.08)	0	0.633
Oceania	3	1.11 (0.98-1.26)	51.9	0.125

#### Meta-analysis for cardiovascular mortality.

**Fig 3:**
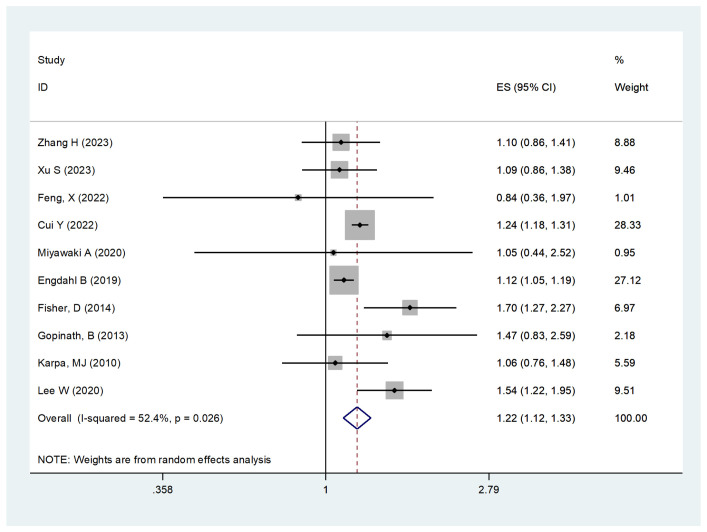
Forest plot for the risk of cardiovascular mortality caused by HL.

Ten studies assessed the relationship between HL and cardiovascular mortality. The pooled results indicated a significant association between HL and increased cardiovascular mortality risk (HR, 1.22; 95% CI, 1.12–1.33; I² = 52.4%, P = 0.000, Fig 3). Sensitivity analysis showed consistent results across studies (S2 Fig).

#### Meta-analysis for cancer mortality.

**Fig 4:**
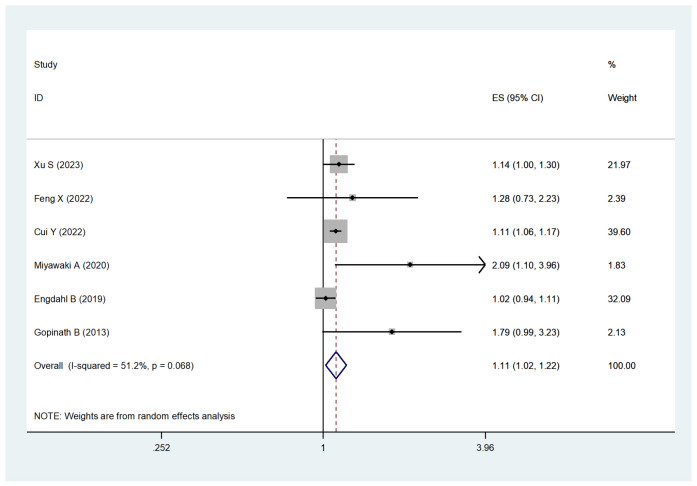
Forest plot for the risk of cancer mortality caused by HL.

Six studies examined the association between HL and cancer mortality. The pooled HR for cancer mortality was significantly elevated in those with HL (HR, 1.11; 95% CI, 1.02–1.22; I² = 51.2%, P = 0.016, Fig 4). Sensitivity analysis confirmed the robustness of these results (S3 Fig).

### Publication bias

**Fig 5:**
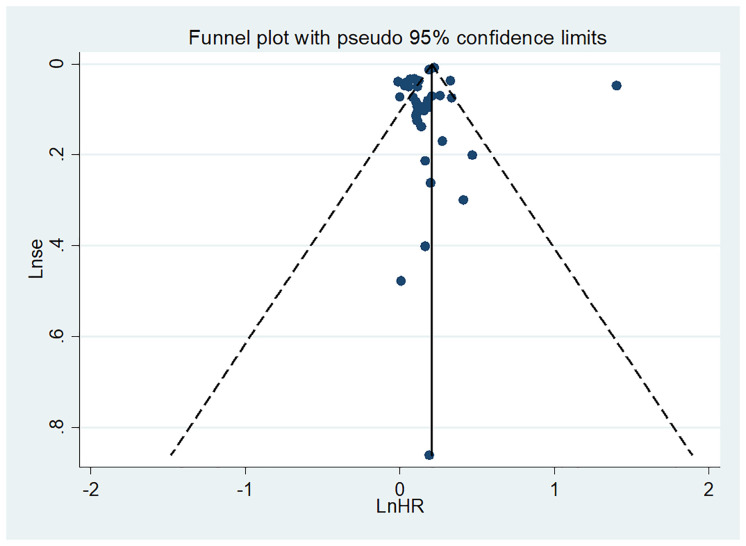
Publication bias of the risk of all-cause mortality caused by HL.

Visual inspection of the funnel plot indicated no significant publication bias for the association between HL and all-cause mortality (Fig 5). Egger’s regression test (P = 0.817) further supported the absence of publication bias. Similar results were observed for the association between HL and cardiovascular and cancer mortality, with no evidence of bias (S4 and S5 Figs).

## Discussion

### Interpretation of findings

The findings highlight HL as a critical yet underrecognized risk factor for mortality, emphasizing the need for integrating auditory health into broader public health strategies. A previous review [20] explored the relationship between HL and mortality, showing that HL increased the risk of all-cause and cardiovascular mortality. However, it did not find a relationship between HL and cancer mortality, identifying only three cohort studies in the analysis. In contrast, our current meta-analysis incorporates more recent studies, providing stronger evidence for the association between HL and cancer mortality. Furthermore, the significant heterogeneity observed in cancer mortality analysis (I² = 51.2%) and the lower bound of the 95% CI approaching 1.02 indicate residual uncertainty in this association, potentially attributable to methodological heterogeneity across studies including variations in cancer endpoint definitions and differential adjustment for critical confounders; consequently, future large-scale cohorts should adopt standardized cancer classification protocols, rigorously adjust for treatment-related confounders, and conduct subtype-specific analyses to elucidate underlying biological mechanisms.

Compared to previous meta-analyses, this study addresses several important gaps, such as incorporating a larger sample size (6.4 million participants), stratifying outcomes by cause-specific mortality, and systematically evaluating methodological and geographic heterogeneity. Earlier reviews predominantly focused on all-cause mortality, with limited exploration of cardiovascular or cancer-related risks. By including 10 studies on cardiovascular mortality and 6 on cancer mortality, our analysis reveals distinct associations, suggesting that HL may influence different disease pathways in specific ways. Moreover, the stronger effect size for audiometric HL compared to self-reported HL underscores the value of objective measurement in accurately capturing biological risks. These advances provide clinicians and policymakers with valuable insights, emphasizing the importance of routine HL screening in health assessments and targeted interventions (e.g., hearing aids, social support programs) to mitigate the associated mortality risks.

The relationship between HL and mortality may be mediated through several interconnected pathways. First, untreated HL often leads to social isolation and reduced environmental awareness, both of which are associated with neuroendocrine dysregulation (e.g., elevated cortisol levels) and chronic inflammation, as demonstrated in studies linking HL to elevated C-reactive protein and interleukin-6 levels [49,50]. Second, auditory processing deficits increase cognitive load, which may exacerbate systemic stress, impair cardiovascular function, and contribute to the development of atherosclerosis [51,52]. Third, shared risk factors such as vascular dysfunction and oxidative stress may underlie both HL and cancer progression, as evidenced by preclinical models demonstrating noise-induced cochlear damage and DNA repair deficiencies [53]. Additionally, research has indicated that autoimmune conditions such as celiac disease are associated with an increased prevalence of sensorineural hearing loss (SNHL). Potential mechanisms underlying this association include autoimmune-mediated attacks targeting inner ear structures, deposition of immune complexes, T lymphocyte-mediated cytotoxicity, and vasculitis affecting the cochlea. These processes may be driven by shared genetic susceptibility leading to a chronic inflammatory state. In celiac disease, anti-tissue transglutaminase antibodies might also contribute to neurological damage, including auditory pathways. Although often subclinical, such hearing impairment could signify early neural involvement and may progress with disease duration. Therefore, in some individuals, hearing loss could serve as an early indicator of underlying autoimmune or inflammatory processes, which have been linked to broader systemic complications and potentially increased mortality [54]. These mechanisms align with our findings, suggesting that HL serves both as a contributor to and a marker of multisystemic dysfunction.

We found that the risk of all-cause mortality was higher in Asia compared with other continents when performing subgroup analysis by continent. Based on the analysis of 13 Asian studies (7 Chinese, 3 Korean, 2 Japanese, 1 Indian), we propose the following evidence-supported hypotheses for this regional disparity:the concentration of studies in rapidly industrializing East Asian nations (China/Korea/Japan: 12/13 studies) aligns with WHO reports on escalating occupational noise hazards in developing economies. Manufacturing sector growth has increased exposure to chronic industrial noise (>85 dB), causing cumulative cochlear damage [55]. Industrial development generates concomitant air pollution and green space reduction, established environmental risk factors for hearing loss in extant literature [56]. Concurrently, East Asia faces accelerating population aging that compounds hearing loss burdens [57]. Inadequate screening programs and insufficient hearing protection measures collectively compound the severity of hearing loss and related disabilities, these systemic deficiencies may elevate all-cause mortality risk through multiple pathways.

### Strengths and limitations

This study’s strengths include its adherence to PRISMA guidelines, extensive subgroup analyses (e.g., stratification by continent and study design), and the inclusion of a diverse range of populations spanning 30 years of research. However, there are limitations, such as residual confounding (e.g., unmeasured socioeconomic factors), high heterogeneity in some subgroups (I² > 95%), and reliance on observational data, which limits the ability to infer causality. Additionally, the predominance of studies from Asia and North America may limit the generalizability of the findings to other regions. Future research should focus on longitudinal studies with mechanistic designs and randomized trials to evaluate the impact of HL interventions on mortality. Furthermore, our findings are not generalizable to children, as no eligible studies addressed childhood hearing loss and mortality. Future research should prioritize longitudinal cohort studies specifically designed to examine the long-term mortality implications of pediatric hearing impairment, particularly through population-based registries linking early-life audiometric data to mortality registries.

## Conclusion

This meta-analysis provides compelling evidence that hearing loss is independently associated with increased risks of all-cause, cardiovascular, and cancer mortality. The stronger associations observed with audiometric assessments and in Asian cohorts highlight the need for standardized diagnostic criteria and region-specific prevention strategies. Clinicians should consider HL not only as a sensory impairment but as a potential marker of broader systemic health deterioration, advocating for early screening and multidisciplinary management.

## Supporting information

S1 Table The retrieval strategies and retrieval results of each database. (DOCX)

S2 Table Reasons for excluding studies from the final analysis after full text assessment. (DOCX)

S3 Table The quality assessment of cohort studies. (DOCX)

S1 Fig Sensitivity analysis of the risk of all-cause mortality caused by HL. (TIF)

S2 Fig Sensitivity analysis of the risk of cardiovascular mortality caused by HL. (TIF)

S3 Fig Sensitivity analysis of the risk of cancer mortality caused by HL. (TIF)

S4 Fig Publication bias of the risk of cardiovascular mortality caused by HL. (TIF)

S5 Fig Publication bias of the risk of cancer mortality caused by HL. (TIF)
