# Supplementary material for: Hearing loss and its association with all-cause and cause-specific mortality: A meta-analysis of cohort studies
Source: PLoS One. 2025 Oct 9;20(10):e0333125. doi: 10.1371/journal.pone.0333125 (PMC12510559; doi:10.1371/journal.pone.0333125)
Supplement: S1 Table — (DOCX) [file pone.0333125.s001.docx]

S1 Table The retrieval strategies and retrieval results of each database

| PubMed | Content | | Result |
| --- | --- | --- | --- |
| #1 | "Hearing Loss"[Mesh] | | 81,624 |
| #2 | (((Hearing Loss*[Title/Abstract]) OR (Hearing Impairment[Title/Abstract])) OR (Hypoacusis[Title/Abstract])) OR (Transitory Deafness*[Title/Abstract]) | | 70,682 |
| #3 | ("Hearing Loss"[Mesh]) OR ((((Hearing Loss*[Title/Abstract]) OR (Hearing Impairment[Title/Abstract])) OR (Hypoacusis[Title/Abstract])) OR (Transitory Deafness*[Title/Abstract])) | | 111,965 |
| #4 | "Mortality"[Mesh] | | 436,221 |
| #5 | ((((Mortalit*[Title/Abstract]) OR (Death*[Title/Abstract])) OR (Case Fatality Rate*[Title/Abstract])) OR (Age-Specific Death Rate*[Title/Abstract])) OR (Crude Death Rate*[Title/Abstract]) | | 1,989,046 |
| #6 | ("Mortality"[Mesh]) OR (((((Mortalit*[Title/Abstract]) OR (Death*[Title/Abstract])) OR (Case Fatality Rate*[Title/Abstract])) OR (Age-Specific Death Rate*[Title/Abstract])) OR (Crude Death Rate*[Title/Abstract])) | | 2,201,727 |
| #7 | "Risk"[Mesh] | | 1,452,063 |
| #8 | Risk*[Title/Abstract] | | 3,261,972 |
| #9 | ("Risk"[Mesh]) OR (Risk*[Title/Abstract]) | | 3,764,381 |
| #10 | ((("Hearing Loss"[Mesh]) OR ((((Hearing Loss*[Title/Abstract]) OR (Hearing Impairment[Title/Abstract])) OR (Hypoacusis[Title/Abstract])) OR (Transitory Deafness*[Title/Abstract]))) AND (("Mortality"[Mesh]) OR (((((Mortalit*[Title/Abstract]) OR (Death*[Title/Abstract])) OR (Case Fatality Rate*[Title/Abstract])) OR (Age-Specific Death Rate*[Title/Abstract])) OR (Crude Death Rate*[Title/Abstract])))) AND (("Risk"[Mesh]) OR (Risk*[Title/Abstract])) | | 746 |
| Embase | Content | | Result |
| #1 | ('hearing'/exp OR hearing) AND ('loss'/exp OR loss) | | 89,402 |
| #2 | 'hearing loss*':ab,ti OR 'hearing impairment':ab,ti OR 'hypoacusis':ab,ti OR 'transitory deafness*':ab,ti | | 84,744 |
| #3 | #1 OR #2 | | 98,394 |
| #4 | mortality | | 2134,471 |
| #5 | 'mortalit*':ab,ti OR 'death*':ab,ti OR 'case fatality rate*':ab,ti OR 'age-specific death rate*':ab,ti OR 'crude death rate*':ab,ti | | 2865,637 |
| #6 | #4 OR #5 | | 3273,828 |
| #7 | risk | | 5472,089 |
| #8 | 'risk*':ab,ti | | 4646,577 |
| #9 | #7 OR #8 | | 5646,907 |
| #10 | #3 AND #6 AND #9 | | 1234 |
| Cochrane Library | Content | Result | |
| #1 | MeSH descriptor: [Hearing Loss] this term only | 4,493 | |
| #2 | (Hearing Loss*):ti,ab,kw OR (Hearing Impairment):ti,ab,kw OR (Hypoacusis):ti,ab,kw OR (Transitory Deafness*):ti,ab,kw | 5,462 | |
| #3 | #1 or #2 | 5,973 | |
| #4 | MeSH descriptor: [Mortality] this term only | 131,145 | |
| #5 | (Mortalit*):ti,ab,kw OR (Death*):ti,ab,kw OR (Case Fatality Rate*):ti,ab,kw OR (Age-Specific Death Rate*):ti,ab,kw OR (Crude Death Rate*):ti,ab,kw | 190,205 | |
| #6 | #4 or #5 | 193,847 | |
| #7 | MeSH descriptor: [Risk] this term only | 326,395 | |
| #8 | (Risk*):ti,ab,kw | 330,861 | |
| #9 | #7 or #8 | 338,433 | |
| #10 | #3 and #6 and #9 | 524 | |
