# Supplementary material for: Hearing loss and its association with all-cause and cause-specific mortality: A meta-analysis of cohort studies
Source: PLoS One. 2025 Oct 9;20(10):e0333125. doi: 10.1371/journal.pone.0333125 (PMC12510559; doi:10.1371/journal.pone.0333125)
Supplement: S2 Table — (DOCX) [file pone.0333125.s002.docx]

S2 Table Reasons for excluding studies from the final analysis after full text assessment

| **NO.** | **Reference** | **Reason for exclusion** |
| --- | --- | --- |
| 1 | Choi, Y., et al., Hearing impairment increases the risk of hip fracture-related mortality and recurrent hip fractures: A propensity score matching analysis. Arch Gerontol Geriatr, 2024. 127: p. 105548. | Irrelevant outcomes |
| 2 | Chen, H., et al, Progression and trajectory network of age-related functional impairments and their combined associations with mortality. iScience, 2023. 26(12): p. 108368. | Irrelevant outcomes |
| 3 | Zhang, X, et al, Association between dual sensory impairment and risk of mortality: a cohort study from the UK Biobank. BMC geriatrics, 2022. 22(1): p. 631. | Irrelevant outcomes |
| 4 | Martz, E., et al., Tinnitus, Depression, Anxiety, and Suicide in Recent Veterans: A Retrospective Analysis. Ear Hear, 2018. 39(6): p. 1046-1056. | Irrelevant outcomes |
| 5 | Honeybrook, A., et al., Hearing and Mortality Outcomes following Temporal Bone Fractures. Craniomaxillofacial Trauma and Reconstruction, 2017. 10(4): p. 281-285. | Irrelevant exposures |
| 6 | Michikawa, T., Prevalence, adverse health, and risk factors in association with sensory impairments: data from a prospective cohort study of older Japanese. Environ Health Prev Med, 2016. 21(6): p. 403-409. | Irrelevant outcomes |
| 7 | Contrera, K.J., et al., Association of hearing impairment and mortality in the national health and nutrition examination survey. JAMA Otolaryngology - Head and Neck Surgery, 2015. 141(10): p. 944-946. | Not cohort study |
| 8 | Lin, C., et al., Sudden sensorineural hearing loss is correlated with an increased risk of acute myocardial infarction: a population-based cohort study. Laryngoscope, 2013. 123(9): p. 2254-8. | Irrelevant outcomes |
| 9 | Arndt, V., et al., Older workers in the construction industry: Results of a routine health examination and a five year follow up. Occupational and Environmental Medicine, 1996. 53(10): p. 686-691. | Irrelevant exposures |
| 10 | Reuben, D.B., et al., The prognostic value of sensory impairment in older persons. Journal of the American Geriatrics Society, 1999. 47(8): p. 930-935. | Irrelevant outcomes |
| 11 | Friedman, G.D., A.L. Klatsky and A.B. Siegelaub, Predictors of sudden cardiac death. Circulation, 1975. 52(6 Suppl): p. III164-9. | Irrelevant exposures |
| 12 | Anstey KJ, Luszcz MA, Giles LC, Andrews GR. Demographic, health, cognitive, and sensory variables as predictors of mortality in very old adults. Psychol Aging. 2001 Mar;16(1):3-11. | Irrelevant outcomes |
