# Supplementary material for: Hearing loss and its association with all-cause and cause-specific mortality: A meta-analysis of cohort studies
Source: PLoS One. 2025 Oct 9;20(10):e0333125. doi: 10.1371/journal.pone.0333125 (PMC12510559; doi:10.1371/journal.pone.0333125)
Supplement: S3 Table — (DOCX) [file pone.0333125.s003.docx]

**S3 Table The quality assessment of cohort studies**

| Study | Year | Selection | Comparability | Outcome | Total |
| --- | --- | --- | --- | --- | --- |
| Cohort studies (n=36) | | | | | |
| Dement J | 2024 | *** | ** | ** | 7 |
| Choi JS | 2024 | **** | ** | ** | 8 |
| Zhang H | 2023 | *** | ** | ** | 7 |
| Tonelli M | 2023 | *** | ** | ** | 7 |
| Xu S | 2023 | *** | ** | *** | 8 |
| Feng X | 2022 | *** | ** | ** | 7 |
| Cui Y | 2022 | *** | ** | ** | 7 |
| Langballe EM | 2022 | *** | ** | ** | 7 |
| Wang J | 2022 | ** | ** | ** | 6 |
| Kim SY | 2020 | *** | ** | ** | 7 |
| Sun J | 2020 | ** | ** | ** | 6 |
| Han H | 2020 | *** | ** | ** | 7 |
| Zhang Y | 2020 | *** | ** | *** | 7 |
| Miyawaki A | 2020 | *** | ** | ** | 7 |
| Lin HW | 2019 | *** | ** | ** | 7 |
| Engdahl B | 2019 | *** | ** | ** | 7 |
| Amieva H | 2018 | *** | * | ** | 7 |
| Schubert CR | 2017 | **** | ** | ** | 8 |
| Loprinzi PD | 2016 | *** | * | ** | 6 |
| Liljas AE | 2016 | *** | * | ** | 6 |
| Genther DJ | 2015 | *** | ** | *** | 8 |
| Fisher D | 2014 | **** | ** | ** | 8 |
| Gopinath B | 2013 | *** | ** | ** | 7 |
| David Feeny | 2012 | *** | ** | *** | 8 |
| Yamada M | 2011 | *** | ** | * | 6 |
| Agrawal N | 2011 | **** | ** | * | 7 |
| Lopez D | 2011 | *** | ** | * | 6 |
| Karpa MJ | 2010 | *** | ** | ** | 7 |
| Denney JT | 2021 | *** | ** | ** | 7 |
| [Lee](https://pubmed-ncbi-nlm-nih-gov-443.vpnm.ccmu.edu.cn/?sort=date&size=200&term=Lee+W&cauthor_id=32397655) W | 2020 | **** | ** | ** | 8 |
| Liu PL | 2016 | *** | ** | ** | 7 |
| Reuben DB | 1999 | **** | ** | *** | 9 |
| Laforge RG | 1992 | *** | * | * | 5 |
| Lam BL | 2006 | ** | ** | ** | 6 |
| Appollonio I | 1995 | *** | - | ** | 5 |
| Mitoku K | 2016 | ** | ** | ** | 6 |

The NOS scale was used to evaluate the quality of the cohort studies.
