# Supplementary figures and images for: Hearing loss and its association with all-cause and cause-specific mortality: A meta-analysis of cohort studies
Source: PLoS One. 2025 Oct 9;20(10):e0333125. doi: 10.1371/journal.pone.0333125 (PMC12510559; doi:10.1371/journal.pone.0333125)

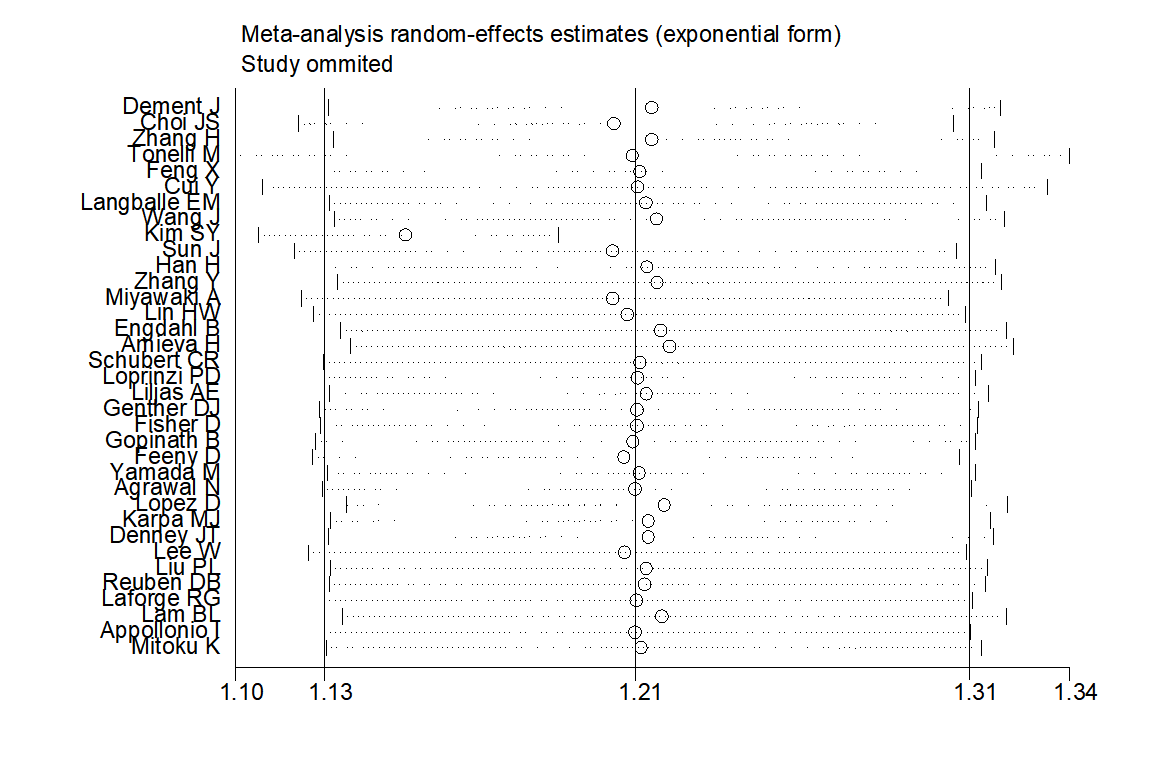

Supplement: S1 Fig — (TIF) [file pone.0333125.s004.tif]

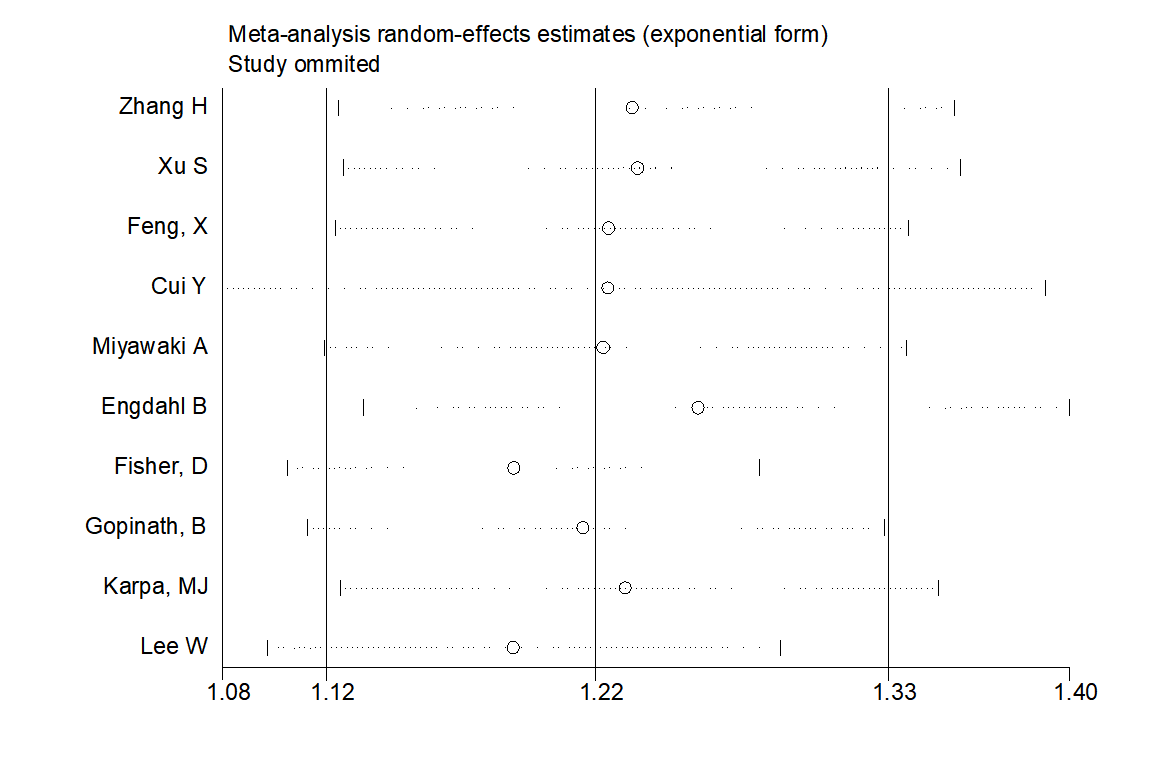

Supplement: S2 Fig — (TIF) [file pone.0333125.s005.tif]

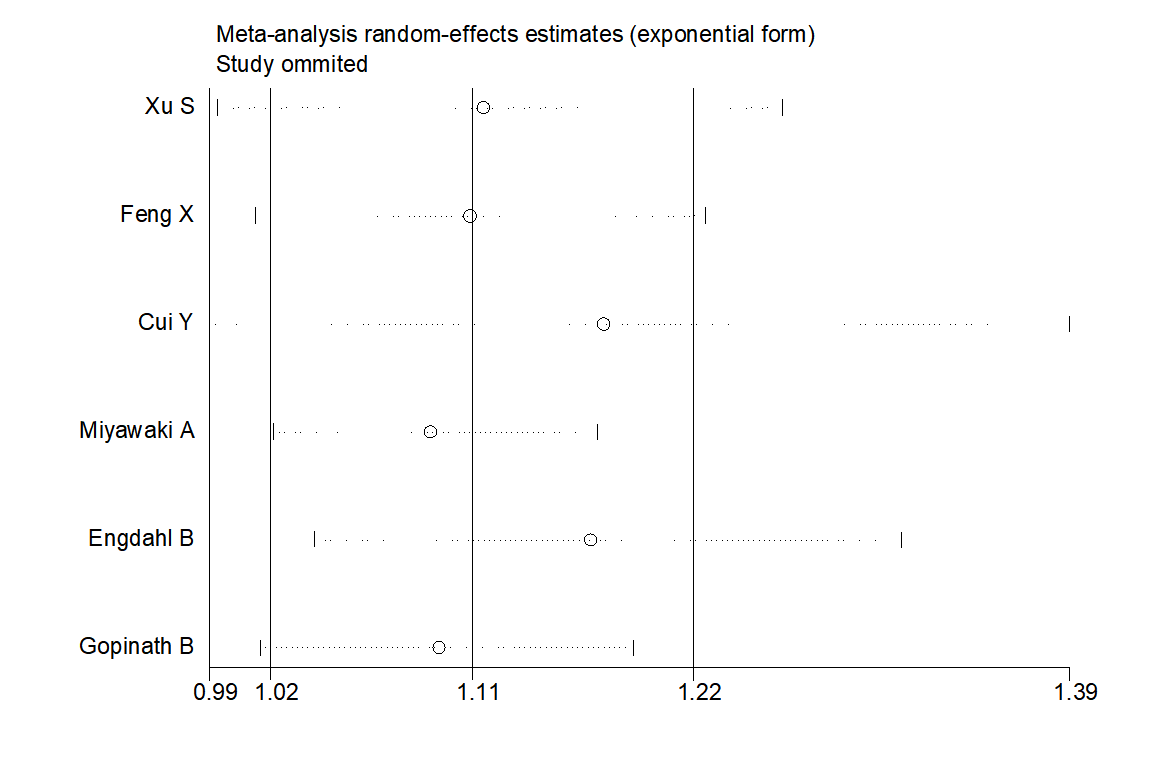

Supplement: S3 Fig — (TIF) [file pone.0333125.s006.tif]

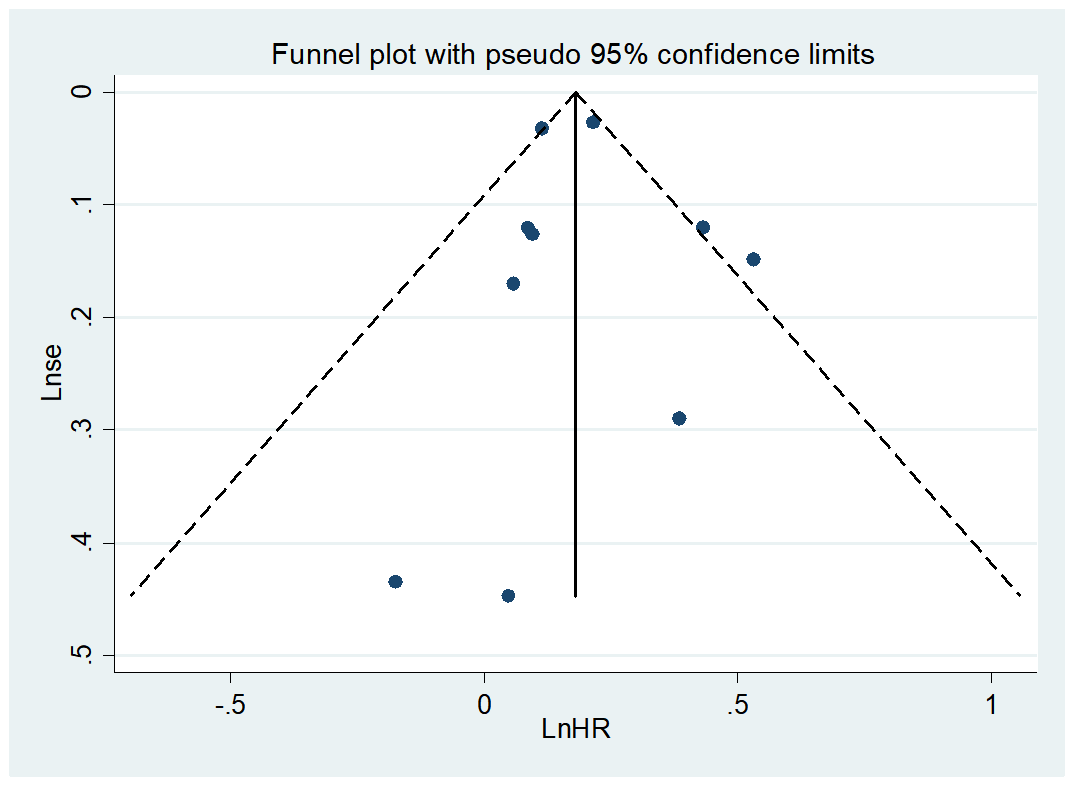

Supplement: S4 Fig — (TIF) [file pone.0333125.s007.tif]

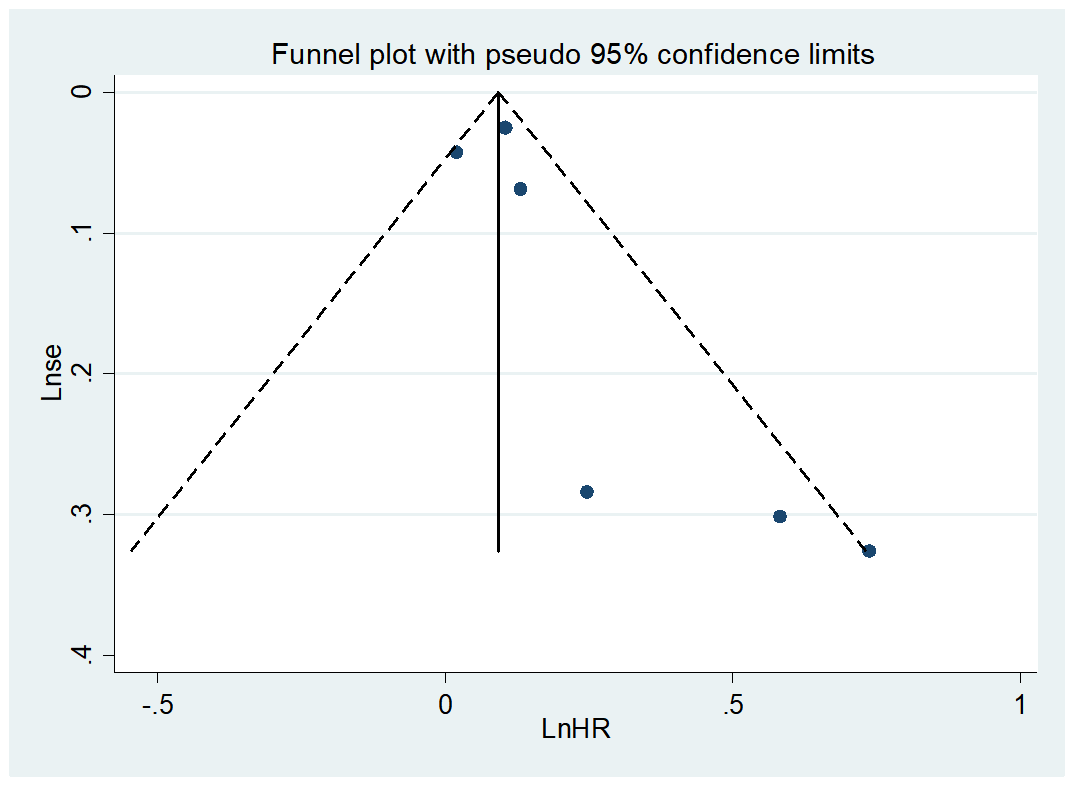

Supplement: S5 Fig — (TIF) [file pone.0333125.s008.tif]
